# The Contribution of Thalamocortical Core and Matrix Pathways to Sleep Spindles

**DOI:** 10.1155/2016/3024342

**Published:** 2016-04-10

**Authors:** Giovanni Piantoni, Eric Halgren, Sydney S. Cash

**Affiliations:** ^1^Massachusetts General Hospital, Boston, MA 02114, USA; ^2^Harvard Medical School, Boston, MA 02115, USA; ^3^University of California, San Diego, La Jolla, CA 92093, USA

## Abstract

Sleep spindles arise from the interaction of thalamic and cortical neurons. Neurons in the thalamic reticular nucleus (TRN) inhibit thalamocortical neurons, which in turn excite the TRN and cortical neurons. A fundamental principle of anatomical organization of the thalamocortical projections is the presence of two pathways: the diffuse matrix pathway and the spatially selective core pathway. Cortical layers are differentially targeted by these two pathways with matrix projections synapsing in superficial layers and core projections impinging on middle layers. Based on this anatomical observation, we propose that spindles can be classified into two classes, those arising from the core pathway and those arising from the matrix pathway, although this does not exclude the fact that some spindles might combine both pathways at the same time. We find evidence for this hypothesis in EEG/MEG studies, intracranial recordings, and computational models that incorporate this difference. This distinction will prove useful in accounting for the multiple functions attributed to spindles, in that spindles of different types might act on local and widespread spatial scales. Because spindle mechanisms are often hijacked in epilepsy and schizophrenia, the classification proposed in this review might provide valuable information in defining which pathways have gone awry in these neurological disorders.

## 1. Introduction

Sleep spindles are characteristic oscillatory events that mark intermediate and deep stages of nonrapid eye movement (NREM) sleep. They arise from the background electroencephalogram (EEG) as oscillations of 0.5–3 s duration with a fundamental frequency between 9 and 16 Hz and reoccur with a rhythmicity of tens of seconds [1, 2]. Spindles have been implicated in multiple functions, from maintenance of sleep depth [3–5] to supporting neural plasticity [6, 7]. These putative mechanisms are thought to underlie the strong link between spindle activity and memory consolidation [8–11].

Spindles are generated within a well-understood circuitry in which the thalamocortical (ThCx) connections play a central role: excitatory ThCx neurons, entrained by inhibitory neurons in the thalamic reticular nucleus (TRN), drive cortical neurons [12–14]. Spindle properties in the time and frequency domains are therefore highly dependent on the thalamic generators and ThCx pathways, although this view does not diminish the role that corticothalamic (CxTh) feedback has in shaping the spatiotemporal evolution of spindle activity [15].

At the cortical level, spindles appear to be heterogeneous. One of the most prominent aspects is the difference between faster and slower spindles, depending on the dominant frequency of each spindle oscillation [16–18]. Faster spindles are usually observed in parietal regions, while slower spindles are found in the frontal regions [19–21], although there is considerable overlap in the EEG topography and in the frequency domain between these two extremes [22]. Intracranial recordings in patients with refractory epilepsy that afford higher spatial resolution might provide a solution to this issue. However, two studies leveraging intracranial EEG (iEEG) reported contradictory findings of whether spindle frequency changes linearly as a function of the anterior-posterior axis [23] or whether there exists a discontinuity around the premotor cortex [24]. The presence of a fundamental frequency for each spindle is made murkier by the observation that some spindles show general slowing during their duration [24], probably due to CxTh feedback.

Another major characteristic that differentiates cortical spindles is the degree to which they involve different cortical areas. Studies in both animal models and humans have described spindles as being highly synchronized between multiple cortical regions [14, 15, 25, 26], while more recent findings in patients with iEEG have challenged this interpretation by demonstrating that spindles are isolated events [24, 27–29]. Studies supporting the former or the latter point of view have relied, in most cases, on different recording methods, leaving open the question of whether the recording techniques might favor one or the other interpretation. In fact, very few studies have compared directly the spatial extent of local and widespread spindles [30, 31].

Crucially, the mechanisms which are responsible for the emergence of local or widespread spindles are unresolved. Two contrasting views suggest that spindle synchronization might occur at the thalamic level, with minimal cortical contribution [34] or as a result of corticocortical (CxCx) interactions [15, 35]. We wondered whether this heterogeneity could be explained in terms of the anatomical differences in the pathways involved in spindle generation.

## 2. Mechanisms of Spindle Generation

The players involved in the generation of spindles have been well characterized thanks to pioneering work in animal studies [12–14]. The major generators are located in the thalamus reflecting in part the reciprocal connections of inhibitory neurons involving TRN and ThCx cells [36–38]. The TRN is a thin envelope of ~1 mm width which encapsulates the frontocaudal length of the thalamus. Neurons in the TRN project, via inhibitory GABA connections, to the major excitatory thalamic nuclei, and most of them express the Ca2+-binding protein parvalbumin [2]. As sleep depth progresses, these neurons begin to fire in bursts, generating massive inhibitory postsynaptic potentials in ThCx neurons [26, 39]. Through deinactivation of the low-threshold Ca2+ channels, ThCx neurons in turn exhibit rebound burst firing which activate the TRN neurons. Interestingly, the TRN is organized in partially overlapping networks which project to specific ThCx neurons [40]. As the thalamus is structured in well-defined nuclei with multiple ThCx pathways, activity in specific TRN subregions can entrain the spindle oscillation in specific cortical areas by driving the corresponding ThCx relay neurons.

Projections from ThCx neurons terminate in two areas: the TRN, where the thalamic loop maintains the balance between excitation and inhibitions of these two groups of neurons, and the cortical regions, where they generate spindle oscillations in cortical field potentials [41]. These cortical potentials can be measured on the scalp and are routinely recorded with EEG and magnetoencephalography (MEG) in humans, where the mechanisms underlying spindle generation are thought to mirror those described in animal studies [2].

The specific contributions of ThCx drive and CxTh feedback to the generation of spindles have been heavily debated [41]. While thalamic regions are sufficient for the emergence of spindle oscillations, as demonstrated by decortication in vivo [38, 42] and recordings in thalamic slices [43], there is strong evidence that the cortex plays a central role in spindle organization [12, 44, 45]. At the minimum, the neocortex can be described as an amplifier of thalamic oscillations [46]. Accumulating evidence indicates that the cortex directly affects the spatial and temporal synchronization of spindle oscillations [12]. In the temporal domain, spindle initiation is associated with a specific phase of the slow waves, which are predominantly cortical events [21, 47, 48]. Furthermore, slow waves triggered by purely cortical stimulation might favor the emergence of spindles during their up state [49]. Conversely, CxCx interaction and cortical feedback can accelerate spindle termination [50].

In summary, while the TRN plays the central role of pacemaker in the generation of the spindle oscillation, the reciprocal connections between excitatory and inhibitory neurons in the thalamus and between the ThCx and CxTh neurons are essential in defining the spatiotemporal dynamics of spindle activity. However, the classical description of spindle generation glosses over the issue of spatial extent and leaves unexplained the causes for the heterogeneity in spindle activity. We propose that these issues can be resolved by adding the spatial characteristics of the ThCx projections to the classical model of spindle generation. Therefore, we need to turn our attention to these pathways and how they affect the spatial extent of spindle oscillations.

## 3. Core and Matrix Pathways

**Figure 1:**
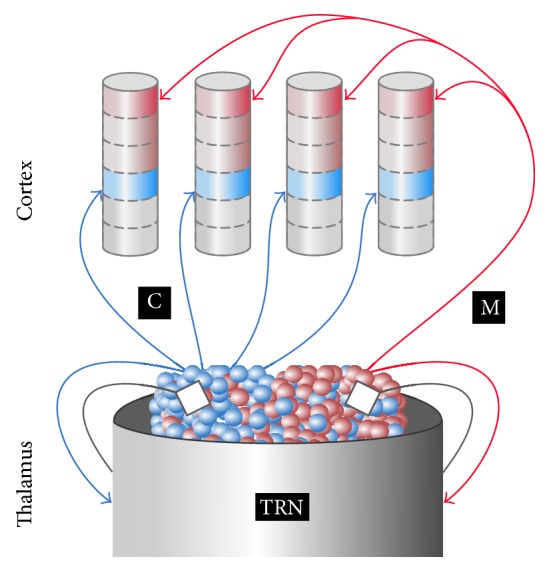
Schematic representation of the core and matrix pathways. Spindles are generated in the reciprocal connections between TRN (thalamic reticular nucleus, in gray) and the thalamocortical neurons, belonging to either the core (in blue, labeled with “C”) or the matrix (in red, labeled with “M”) pathway. Neurons belonging to the core and matrix pathways are mostly intermixed inside the thalamus, but individual nuclei might have prevalence of either type. The projections from the TRN are inhibitory, indicated by the square terminals. The core pathways are independent of each other, project to a single brain area, and reach the middle, granular layers of the cortical column. The matrix pathways have a broader and more diffuse fan-out and target more superficial, supragranular layers.

Because of the central role of the thalamus in spindle generation and its synchronization, the electrophysiological properties and connectivity patterns of the thalamic nuclei have a strong influence on the spindle properties. With the exception of the TRN, which targets other thalamic nuclei, the thalamus is composed of neurons that project to cortical and subcortical areas [51]. ThCx neurons can be classified based on the cortical target of their projections. Two major pathways have been identified: the core (also called C-type) pathway and the matrix (also called M-type) pathway (Figure 1) [32, 52]. These two pathways differ in a number of ways [33]. Core neurons are spatially selective and topographically organized, target a single cortical area, and project mostly to the granular layer, the major input layer of the cortex. Matrix neurons have diffuse, multiarea projections, characterized by multiple distant arbors, and reach mostly superficial layers of the cortex. The distribution of core and matrix neurons is not uniform across thalamic nuclei: nuclei most strongly associated with sensory relay, such as the lateral geniculate body, are dominated by core neurons, while some other nuclei lack those neurons completely [32, 53]. Although tracing techniques have been essential in identifying these two pathways, there are still unresolved issues regarding the connections within and between thalamic nuclei. In particular, it is not clear whether core and matrix neurons have direct, reciprocal connections and whether TRN neurons project more strongly to either of the two neuron types.

Based on their anatomical connections, these two pathways have been assigned specific functional roles in the thalamocortical dialogue. Core projections are those usually associated with relay neurons, which transmit sensory information to cortical areas. These are the most intensively studied type of thalamic neuron. The matrix neurons, on the other hand, are thought to maintain the overall state of the cortex, to drive the internal excitability of large cortical regions, and to control and time their propensity to respond to inputs [54]. Conversely, these thalamic neurons are affected by a strong CxTh feedback [52]. The two ThCx pathways reflect the dual nature of the thalamus as a whole, which integrates two different roles. The first role is the one in which sensory information is passed through relatively faithfully to cortical regions, albeit amplified or suppressed based on attentional mechanisms. The second role is to maintain the state of the cortex and to support an appropriate degree of activation, in either wakefulness or sleep.

## 4. Core and Matrix Spindles

The importance of the thalamus and ThCx connections in entraining the emergence of cortical spindles and the major organizational principle of the ThCx pathways, which subdivides them in core and matrix projections, provide grounding for the hypothesis that spindles that occur in either the core or the matrix neurons will differ based on the anatomical characteristics of these two pathways. According to this hypothesis, spindles that arise from the interaction of TRN and core neurons will have a highly specific and local cortical profile, while spindles that arise from the interaction of TRN and matrix neurons will have more diffuse and spatially synchronized cortical distribution. An important aspect of this hypothesis is that the two types of spindles will differ in virtue only of the cortical terminations of the core and matrix ThCx neurons and not because of any electrophysiological difference, such as firing rate or firing threshold, of these neuronal populations. Therefore, recordings in the decorticated thalamus will not be able to distinguish these two types of spindles; differences will only emerge at the cortical level. This interpretation dovetails with the underlying assumption that both neuron types are equally likely to generate spindles, as, to the best of our knowledge, there is no evidence showing that one of the two neuronal types is more or less involved in spindle activity.

**Figure 2:**
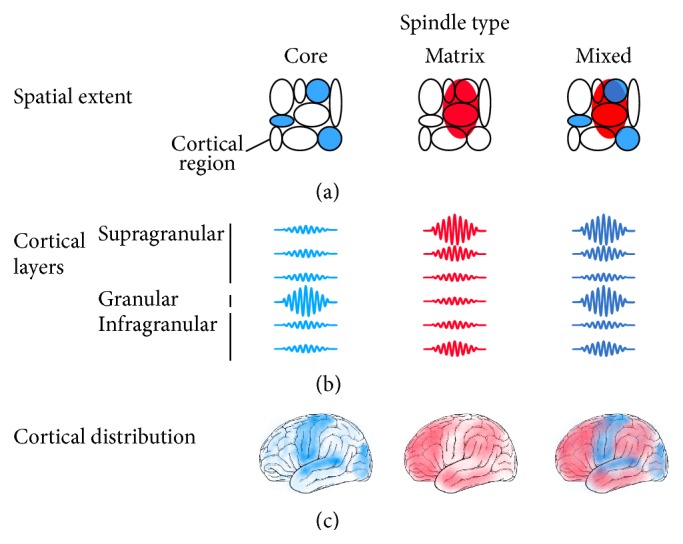
Predictions of the hypothesis that spindles can be classified in core or matrix spindles, depending on the ThCx pathways involved in their generation. In addition to these two extreme types, we expect that most spindles will have mixed properties of either spindle type. (a) The first prediction of this hypothesis is that core spindles are limited to single cortical regions, while one matrix spindle affects multiple brain regions simultaneously. We expect to observe independent core spindles in multiple regions, while matrix spindles are not strictly limited by the boundaries of the cortical regions. (b) The second prediction is that the laminar distribution differs between core and matrix spindles. Core spindles are expected to be most prevalent in granular layers, which receive ThCx afferents from thalamic relay neurons, while activity of matrix spindles is largest in superficial layers and in layers receiving diffuse thalamic input. Mixed spindles should have spindle activity in all the layers or show a temporal evolution from spindle activity being dominant in some layers in the first cycles and in other layers in later cycles. (c) As core ThCx pathways tend to arise from relay thalamic nuclei [32, 33], we expect to find most core spindles in primary sensory and motor cortices. Neurons projecting to the matrix ThCx pathways, on the other hand, are more diffuse throughout the thalamus, suggesting that matrix spindles might occur in most cortical regions.

The fundamental idea is that the characteristics of the underlying ThCx pathways will have a corresponding effect on the characteristics of the cortical spindles. This interpretation has clear and testable predictions that have only partially been explored (Figure 2). As the core and matrix pathways differ in cortical extent, we expect that there could be the two types of spindles which have more local or more widespread synchronization profiles. Because these two pathways target separate cortical layers, we expect that core spindles will be more prominent in granular layers and that matrix spindles will be more prominent in superficial layers. As core pathways dominate in primary sensory areas, we expect to observe more local and spatially limited spindles in the postcentral, occipital, and posterior temporal regions.

Although they are useful in conceptualizing the major differences between core and matrix spindles and in guiding research investigations, these predictions should not be taken as absolute. There are a few qualifications based on known anatomical and electrophysiological properties that should be made explicit. The first aspect is that the core and matrix spindles should not be considered as mutually exclusive. It is easily conceivable that a few cycles within a spindle might be generated in one or multiple core pathways before activation of the matrix pathway, so that a spindle might have highly local characteristics in the first cycles and more widespread synchronization later. In fact, we expect that the majority of spindles will consist of a mixture of core and matrix properties at different stages of their temporal evolution. The second aspect is that some matrix pathways can be very localized, such as M-type neurons that have been further divided in local and multiareal M-type neurons [53]. These local matrix neurons target superficial layers, like the multiareal neurons, but their arborization is very limited, consisting of single or few arbors reaching a single area, similarly to core neurons. This finding blurs the distinction between core and matrix neurons but does not affect the underlying hypothesis that the anatomical connections of the ThCx pathways will affect the dynamic properties of the cortical spindles.

The third qualification is that although the ThCx connections play a central role in shaping spindle characteristics, the CxTh feedback is essential in determining spindles' synchronization properties [15] and spatiotemporal evolution [35]. Cortical activity propagates to the thalamus, especially its relay nuclei, through two pathways: one modulatory CxTh input from cortical layer VI and one tightly localized input from cortical layer V [55, 56]. This CxTh activity in turn projects back to other parts of the cortex, representing therefore a thalamic pathway for CxCx interaction [57]. Accordingly, the CxTh modulation provided by these two pathways might induce stronger CxCx coherence of spindles, especially for those arising from the core ThCx projections, and might represent a complimentary mechanism of spindle synchronization, in addition to the core and matrix ThCx connections.

Based on the premise that the anatomical substrate of the ThCx projections affects the spatial characteristics of spindles, we propose that spindles whose activity is influenced by matrix or core pathways will differ in terms of spatial extent and, more generally, of spatiotemporal evolution. We will examine the differences between these two types of spindles in an explicit computational model, in laminar recordings in animals, and in human studies combining EEG and MEG.

### 4.1. Computational Model

A highly constrained computational model of these two types of spindles can improve our understanding of the specific predictions that derive from the hypothesis. The ThCx circuitry involved in the spindle generation has been well described and therefore it presents a valuable case for the development of computational models that replicate the essential features of spindle activity. Multiple computational models of the ThCx and CxTh role in spindle activity have been developed [12, 50, 58–60]. One model has incorporated the two types of pathways in the generation of spindle and has modeled the expected differences between core and matrix spindles [61].

As in previous work, spindle generation was initiated by the reciprocal connections between inhibitory TRN neurons and excitatory ThCx neurons. Excitatory ThCx neurons were modeled as either core or matrix whose properties were identical except that the neurons belonging to the matrix pathway had up to five times wider fan-out of the cortical projections than those belonging to the core pathway, in accord with previous anatomical descriptions of the primate ThCx connections [62].

Bonjean et al. [61] found that the initial cycles were similar in the core and matrix pathways but the spatial propagation became more synchronous in the matrix pathway than in the core pathway as the spindle unfolded. The termination process was also different in the two pathways: core spindles decayed asynchronously across multiple locations, while matrix spindles had a temporally defined offset. This effect was in part due to the CxTh feedback projections that influence the matrix neurons more than the core neurons [50, 63]. A crucial observation was that the spindles often involved both pathways: spindles which were generated in the core pathway were more likely to spread to the matrix pathway after a few hundred ms than to the opposite direction.

By explicitly modeling the assumptions underlying the hypothesis presented here, this work confirms the prediction that the cortical fan-out of the two pathways can affect the spatial extent of the synchronization of core and matrix spindles. It also provides a framework for the dynamic interaction between these two pathways, often within the same spindles, where the first cycles might involve only one of the two pathways and where the termination might or might not involve CxTh feedback.

### 4.2. Laminar Profiles

Based on the laminar projections of the core and matrix pathways, we expect that the two types of spindles can be dissociated based on the cortical layers that show the largest activity. The thalamocortical core pathway terminates mostly on the granular layer, while the matrix pathway targets more superficial layers. These predictions have not been explicitly tested to the best of our knowledge.

Two classical studies have investigated spindle oscillations using laminar probes and their results might shed some light on this distinction [46, 64]. Both of these studies reported two types of spindles, although this distinction was not framed in terms of core/matrix spindles. The major observation of Kandel and Buzsáki [46] is that spindles differ in the location of the sources and of the sinks across cortical layers. The more commonly observed spindles had sinks in layer IV, while they reported other spindles that had a more superficial profile. Intriguingly, both studies find that spindles might have either of the two laminar profiles, or more often a single spindle oscillation might evolve from one type to the other.

These classical laminar studies support several aspects of the hypothesis presented in this review. The first aspect is the usefulness of using laminar profiles to classify spindles in two major groups. The second aspect is that while some spindles have largest sinks in the granular layer IV, other spindles have significant activity in superficial layers. Thirdly, these studies indicate that most spindles consist of a mixture of the two spindle types. Considering the ability of laminar recordings to identify spindle types, further studies employing this technique will be useful in investigating the temporal evolution of spindle oscillations across cortical layers and between spindle types.

### 4.3. EEG versus MEG

The distinction between core and matrix is particularly useful in explaining the intriguing observation that while spindles observed on scalp EEG have high interregional coherence, spindles measured with MEG showed high variability across cortical areas, with asynchronous phase and amplitude across sensors and with poor correlation to the simultaneously recorded EEG [30, 65]. Multiple measures supported this conclusion. Coherence between pairs of EEG electrodes during spindle activity was 0.7, while coherence between pairs of MEG sensors was 0.3. Spindles in the MEG were spatially much more variable. Techniques, such as principal component analysis (PCA), can be used to summarize the amount of variance inherent in the recordings during sleep. 50% of the variance in the EEG spindle recordings was explained by two principal components, indicating that the signal was relatively simple. On the other hand, to account for the same variance, more than 15 PCA components were necessary for the MEG spindle signal [65]. Correspondingly, the estimated sources vary between the two recording techniques: while sources computed from spindle recordings in the EEG were widely distributed and synchronous across the cortex, sources computed from the MEG signal were highly specific, with the phase of oscillation varying between multiple locations. In contrast to the EEG source analysis, MEG source estimates indicate that sleep spindles are generated by multiple brain regions relatively independently [30, 66].

These observations are in agreement with recordings that combined scalp EEG with iEEG. The spindles observed in the EEG were poorly synchronized with spindles captured with depth electrodes, suggesting that possibly different mechanisms are involved in the generation of spindles appearing in one or the other recording system [28].

These results integrate well with the framework where MEG is more sensitive to core spindles and EEG spindles might pick up better the matrix spindles that occur mostly in superficial cortical layers. This might be the result of biophysical differences in the orientation of the cortical neurons that are entrained in the spindle frequency, as the magnetic signal is highly dependent on the orientation of the dipole source [67]. This interpretation would also explain the small delay observed between EEG and MEG [68]; we can interpret this as spindle starts as core and then becomes matrix thanks to CxTh feedback.

Current EEG/MEG studies do not provide unequivocal evidence supporting the prediction that core spindles might occur more likely over primary sensory areas, while matrix spindles might favor associative areas. The identification of possible differences in the cortical distribution of the different types of spindles is complicated by the very low spatial coherence of MEG spindles, which results in multiple, sparse sources [30, 66]. Nevertheless, a pattern seems to emerge in which MEG spindles are mostly localized over precentral areas including primary motor cortex and postcentral areas of the parietal cortex of each hemisphere [66]. On the other hand, a similar study was not able to unequivocally identify the distinctive generators of EEG and MEG spindles [30]. In addition, significant functional Magnetic Resonance Imaging (fMRI) activity associated with EEG spindles was observed in paralimbic areas, in prefrontal regions, and also in the vicinity of auditory cortices, in addition to the thalamus [69]. Overall, this lack of a substantial difference in cortical distribution between EEG and MEG spindles in the literature calls for further studies necessary to test the prediction of differential cortical areas involved in core and matrix spindles.

## 5. Outstanding Questions

The experimental observations described above provide some supporting evidence for the hypothesis that core and matrix spindles are affected by the terminations of their respective ThCx pathways. The hypothesis, however, will require further research to fully flesh out its implications to the field of neuroscience. Below we outline some major issues that need particular attention.

### 5.1. Heterogeneity of Spindle Oscillations

A consistent finding reviewed in Introduction is the heterogeneity of cortical spindles. The hypothesis put forward in this study indicates a possible mechanism underlying this heterogeneity. A major issue in the spindle literature that has become central in the past few years is the challenge that more recent studies, often based on iEEG, have contradicted the classical notion that spindles are highly synchronous throughout the cortex [24, 27, 28]. The distinction between core and matrix spindles offers a possible solution to these opposing views. According to this hypothesis, the distinction between core and matrix spindles reflects the distinction between local and widespread events. This implies that spindles are not exclusively local, but spindle oscillations might be synchronous within a cortical area or across multiple areas depending on the underlying ThCx pathways involved in their generation.

The prevalence of local or widespread spindles in a specific study might therefore be the consequence of recording techniques that are more sensitive to the properties of core or matrix spindles. In particular, the local properties of spindles in iEEG studies might be explained by the fact that this technique, similarly to MEG, is most sensitive to core spindles. This would account for the unexpectedly low synchrony between iEEG and EEG spindles [28], which reflects the low correspondence between MEG and EEG spindles [65]. Other factors might be at play in animal studies. Core and matrix ThCx pathways can be traced using sensitive labeling techniques [53], suggesting that neurons in these two pathways might react differently to anesthetic and pharmacological agents. Therefore, the application of a particular drug might have the unintended consequence of silencing a specific ThCx pathway. Attention is required in further studies in choosing substances that minimize any side effect to the complex dynamics involved in spindle generation.

A possible dissociation between core and matrix spindles might be found in their sensitivity to circadian rhythms and general arousal levels. It has been shown that the occurrence of spindles depends on the circadian clock [70, 71], indicating that at least some spindles are influenced by the modulation of subcortical brain nuclei. Because the matrix ThCx pathways are involved in maintaining the state of the cortex, it is conceivable that spindles generated within this pathway are more sensitive to general arousal level, which are partly affected by circadian rhythms.

Whether the dichotomy of core/matrix spindles is reflected in other classifications that have been applied to spindles, such as fast/slow spindles, remains an open question. To the best of our knowledge, there is no evidence that indicates correspondence in the resonance frequency of the spindle oscillations and the degree to which it is either focal or widespread. There is also no known difference in resonance frequency for the core versus matrix pathways which would suggest that such a dichotomy would be expected. A confounding factor is the poor mapping of fast and slow spindles between human and animal studies. Because most works on the anatomy of core/matrix ThCx projections have been conducted in animals, it is necessary to be cautious when applying findings in animal models to the human brain as the frequency ranges used in animals and humans might not be directly comparable.

### 5.2. Plasticity and Memory Consolidation

The difference in the spatial properties between core and matrix spindles has several functional implications. The temporal dynamics associated with spindle oscillations are known to favor neural plasticity, by promoting short-term synaptic homeostasis [14, 72] and inducing NMDA-dependent long-term potentiation [7], gene expression [6], and spike-timing-dependent plasticity [73]. These mechanisms are thought to underlie the intimate link between spindles and memory consolidation [2, 74–76]. While pioneering work in this field found a topographically unspecific correlation between spindle count and memory improvement and intelligence (reviewed in [18]), the availability of high-density recordings has allowed researchers to identify specific changes in spindle activity in multiple brain regions. Spindles recorded above cortical regions associated with the execution of the task had a stronger correlation with postsleep improvement [77, 78], suggesting that offline memory improvement is highly local and anatomically constrained. When auditory memory cues which had been associated with visual stimuli during task execution were selectively reactivated during sleep, regions involved in visuospatial processing expressed spindles of higher amplitude, indicating network-specific reactivation of cortical memory traces [79]. High spatial specificity of spindle oscillations for category of visual stimuli has been suggested by correlated activity observed in temporal cortices in an fMRI study [80].

Overall, these results indicate that memory consolidation is associated with both a widespread increase and also specific regional changes in spindle activity, depending on the task design and the cortical regions involved in the neural plasticity. We propose that the dual role of spindles in memory consolidation mirrors the distinction between matrix and core spindles. It is conceivable that these two spindle types might affect widespread or spatially constrained cortical areas. While matrix spindles support a general activation of the large cortical regions, core spindles might be implicated in regulating the neural mechanisms underlying local memory processing. This interpretation introduces the role of spindles in neural plasticity as a flexible mechanism that adapts to the cortical needs involved in memory consolidation.

Furthermore, the organization of the ThCx pathways in circumscribed or widespread projections might reflect the functional role associated with each spindle type. As described above, core pathways dominate in primary sensory areas, while matrix connections are more common in regions commonly thought of as belonging to the association cortex. Accordingly, sensory and motor areas might have higher prevalence of core spindles, which, due to the limited fan-out of the ThCx connections, are in a favorable position to induce highly local plasticity within small cortical circuits. This would provide a powerful mechanism to enhance learning of specific sensory and motor skills. On the other hand, association cortices, such as the prefrontal regions, do not require this high level of spatial specificity but instead they often integrate information from multiple distant cortical areas. Based on the anatomy of the ThCx projections, these regions might be expected to be more likely to generate matrix spindles, which are perfectly posed to activate multiple regions and favor memory consolidation across cortical areas [75].

These considerations fit nicely with the recent observation of spindles in the medial temporal lobe, more specifically in the hippocampus. Doubts regarding a possible pathological origin [81, 82] have been dispelled after consistent findings even in nonepileptic hippocampi [28, 29]. Hippocampal spindles are thought to arise from a circuitry involving the TRN and the anterior thalamic nucleus, with which the hippocampus shares reciprocal connections [83], similarly to the ThCx generators involved in neocortical spindles. Because the hippocampus is not organized in six distinct layers, it is unclear whether the classification in core and matrix pathways, and consequently in core and matrix spindles, applies to the allocortex. Hippocampal spindles were found to be more spatially restricted and to have lower synchrony [28], suggesting that very local pathways connect the thalamus to the hippocampus. This highly localized spindle activity might be contrasted with the widespread activation observed in the prefrontal cortex discussed above. These two regions are known to interact during memory encoding and retrieval [84], and the interplay of sleep rhythms during sleep might enhance memory consolidation during sleep [75].

### 5.3. Implications for Brain Disorders

The distinction between core and matrix spindles holds great promise for the study of brain disorders associated with disrupted spindle oscillations. Changes in spindle activity have been observed in several disorders, the two most prominent of which are schizophrenia [85, 86] and some forms of epilepsy [26, 87–89]. It is thought that the same thalamocortical loops that are responsible for spindle generation are hijacked in some forms of epilepsy [26, 90]. In particular, interictal spikes in some primarily generalized epilepsies are thought to arise from a disruption of the same thalamocortical circuits that generate spindles [87, 91].

A deeper understanding of the mechanisms underlying spindle generation will shed light on the thalamic and cortical regions affected by these disorders. Multiple studies investigating the role of sleep in neuropsychiatric disorders have considered the spindle as a phenomenon with a single generative mechanism [85, 86, 92, 93]. The hypothesis put forward here on the other hand presents two partially independent ThCx pathways in the generation of core and matrix spindles. Although currently the evidence is largely indirect, we suggest that some of these disorders might affect only one particular pathway, therefore resulting in abnormal core or matrix spindles. Identification of which spindles are the most affected by a particular disorder might improve the description of the underlying ThCx mechanisms, which will help identify potential treatment targets. Preliminary steps have already been taken in this direction, as in the work of Kandel and Buzsáki [46] comparing the laminar profile of normally occurring spindles with the pathologically high voltage spike-and-wave spindles, a widely accepted animal model of generalized spike-and-wave epilepsy.

## 6. Conclusions

Based on the importance of the ThCx circuits in spindle generation and the anatomical and hodological properties of the core and matrix neurons, we have advanced the hypothesis that the ThCx pathway will affect the spatial extent of spindle oscillations. We propose a classification of core and matrix spindles which can be studied with a variety of techniques, such as EEG/MEG, laminar probes, and computational models. It is important to reiterate that the spindles are very likely to have a mixture of the two extreme types, as we expect that the contribution of each pathway evolves dynamically throughout the course of every spindle. This classification will be useful in guiding further hypothesis-driven investigations into the underlying mechanisms involved in spindle generation and into the functional role of these emblematic oscillations. This future work will help us better understand the cause of disorders affecting spindle oscillations and hopefully will result in the development of therapeutic interventions.

## Abbreviations

CxCx: Corticocortical
CxTh: Corticothalamic
EEG: Electroencephalogram
fMRI: Functional Magnetic Resonance Imaging
iEEG: Intracranial EEG
MEG: Magnetoencephalography
NREM: Nonrapid eye movement
PCA: Principal component analysis
ThCx: Thalamocortical
TRN: Thalamic reticular nucleus.
